# 3D-QSAR, design, molecular docking and dynamics simulation studies of novel 6-hydroxybenzothiazole-2-carboxamides as potentially potent and selective monoamine oxidase B inhibitors

**DOI:** 10.3389/fphar.2025.1545791

**Published:** 2025-01-28

**Authors:** Dong Xie, Yongzheng Tian, Li Cao, Penghang Guo, Zhibiao Cai, Jie Zhou

**Affiliations:** ^1^ Department of Neurosurgery, The 940th Hospital of Joint Logistics Support Force of Chinese People’s Liberation Army, Lanzhou, China; ^2^ Department of First Clinical College of Medicine, Gansu University of Traditional Chinese Medicine, Lanzhou, China

**Keywords:** neuroprotective activity, 6-hydroxybenzothiazole-2-carboxamide, monoamine oxidase B, QSAR, CoMSIA

## Abstract

**Background:**

6-hydroxybenzothiazole-2-carboxamide is a novel, potent and specific inhibitor of monoamine oxidase B (MAO-B), which can be used to study the molecular structure and develop new neuroprotective strategies.

**Objective:**

The aim of this study was to create an effective predictive model from 6-hydroxybenzothiazole-2-carboxamide derivatives to provide a reliable predictive basis for the development of neuroprotective MAO-B inhibitors for the treatment of neurodegenerative diseases.

**Methods:**

First, the compounds were constructed and optimized using ChemDraw and Sybyl-X software. Subsequently, QSAR modeling was performed using the COMSIA method in Sybyl-X to predict the IC50 values of a set of novel 6-hydroxybenzothiazole-2-carboxamide derivatives. The ten most promising compounds were screened based on the IC50 values and tested for molecular docking. Finally, the binding stability and dynamic behavior of these compounds with MAO-B receptors were analyzed by molecular dynamics simulation (MD).

**Results:**

The 3D-QSAR model showed good predictive ability, with a q^2^ value of 0.569, r^2^ value of 0.915, SEE of 0.109 and F value of 52.714 for the COMSIA model. Based on the model, we designed a series of novel 6-HBC derivatives and predicted their IC50 values by the QSAR model. Among them, compound 31.j3 exhibited the highest predicted IC50 value and obtained the highest score in the molecular docking test. MD simulation results showed that compound 31.j3 was stable in binding to the MAO-B receptor, and the RMSD values fluctuated between 1.0 and 2.0 Å, indicating its conformational stability. In addition, energy decomposition analysis revealed the contribution of key amino acid residues to the binding energy, especially Van der Waals interactions and electrostatic interactions play an important role in stabilizing the complex.

**Conclusion:**

In this study, the potential of 6-hydroxybenzothiazole-2-carboxamide derivatives as MAO-B inhibitors was systematically investigated by 3D-QSAR, molecular docking and MD simulations. The successfully designed compound 31.j3 not only demonstrated efficient inhibitory activity, but also verified its stable binding to MAO-B receptor by MD simulation, which provides strong support for the development of novel therapeutic drugs for neurodegenerative diseases. These findings provide important theoretical basis and practical guidance for future drug design and experimental validation.

## 1 Introduction

Neurodegenerative diseases (NDD) are a collection of illnesses marked by the gradual deterioration of nerve cells in either the central nervous system (CNS) or peripheral nervous system (PNS). Parkinson’s disease and Alzheimer’s disease are the most prevalent examples of such conditions. Parkinson’s disease is characterized by the gradual degeneration of neurons, which causes motor system impairments (ataxia). This leads to imbalances, movement problems, and other symptoms such as resting tremor, muscular stiffness, decreased postural reflexes, and difficulty walking. This condition affects a significant number of individuals globally (Wilson et al., 2023). As the world’s population ages, the occurrence of neurodegenerative illnesses is steadily rising each year on a worldwide scale. It affects around 1%–3% of those who are 65 years old or older (Santos García et al., 2019). Presently, there are few effective therapy alternatives available for NDD. Despite the few therapeutic choices available, the success rate remains quite low. To enhance the quality of life and increase the chances of survival for individuals, it is essential to advance the development of novel pharmaceuticals and implement innovative treatment approaches.

Monoamine oxidase (MAO) is situated on the external mitochondrial membrane and consists of two isoforms, MAO-A and MAO-B. MAO-B is mostly located in the brain, platelets, and liver. The overall level of MAO in the brain is estimated to be composed of around 20% MAO-A and 80% MAO-B (Hoy and Keating, 2012; Teo and Ho, 2013). The primary role of MAO-B is to break down phenylethylamine, tryptamine, and methylhistamine by catabolism. MAO is crucial in the breakdown of neurotransmitters, which in turn controls the quantities of monoamines in the brain (Konradi et al., 1987). Heightened concentrations of these enzymes may result in heightened generation of H2O2, which can induce oxidative stress and contribute to various neurodegenerative disorders (Lemke et al., 2019). Nevertheless, the presence of H2O2 causes the oxidation of lipid peroxides and the consequent formation of α-synuclein (α-syn) aggregates. These aggregates are the primary constituents of Lewy bodies (LBs), which in turn contribute to motor impairments in Parkinson’s disease (PD) (Marotta et al., 2021). MAO inhibitors mitigate oxidative stress by suppressing the synthesis of H2O2, hence facilitating the effective control of stroke and tissue damage caused by hydroxyl radicals resulting from hydrogen peroxide generation (Tipton et al., 2004).

Hyperphosphorylation of Tau proteins causes the development of neuronal protofibrillary tangles (NFTs) within neurons, which is a particular change in Parkinson’s disease (PD) (Seitz et al., 2002). Tau is a protein that binds to microtubules and is mostly found in neurons in the brain (Cleveland et al., 1977). MAO-B activity contributes to the generation of Aβ peptides, which in turn leads to tau phosphorylation (Behl et al., 2021). Alpha Synuclein (α-Syn) and tau are significant neuropathogenic proteins that have a crucial function in neurodegenerative disorders (Wilson et al., 2023; Zbinden et al., 2020). Compounds containing MAO-B have shown encouraging outcomes in the treatment of neurodegenerative disorders such as Parkinson’s disease and Alzheimer’s disease (Lu et al., 2013).

At present, a limited number of MAO inhibitors have been approved for commercial use, such as selegiline (R-(−)-deprenyl) and rasagiline, which act in an irreversible manner (Liu et al., 2015), and safinamide, a reversible MAO inhibitor (deSouza and Schapira, 2017). However, these inhibitors are associated with various side effects and limited efficacy. Recent studies have explored novel 6-hydroxybenzothiazole-2-carboxamide derivatives as potential MAO-B inhibitors, showing promising results in terms of IC50 values (Al-Saad et al., 2024). Our study aims to conduct a broader comparison, encompassing not only IC50 values but also molecular docking scores, molecular dynamics simulations, binding free energies, and key amino acid residue contributions, to comprehensively demonstrate the advantages of our newly designed compounds. Significantly, selegiline has been linked to orthostatic hypotension and hallucinogenic adverse effects (Jiang et al., 2020). Safinamide is known to cause birth defects and has also been linked to an increased risk of retinal degeneration. Therefore, it is not recommended to use safinamide during pregnancy or in those with retinal illness (Bette et al., 2018).

6-hydroxybenzothiazole-2-carboxamide and its derivatives, as inhibitors of monoamine oxidase B (MAO-B), exhibit remarkable structural novelty and pharmacological activity. Compared with existing MAO-B inhibitors on the market, such as selegiline and rasagiline, 6-hydroxybenzothiazole-2-carboxamide derivatives feature unique amide substituent modifications in their structures. These modifications not only enhance their selective inhibitory effect on MAO-B but also reduce the risk of side effects. Particularly, by introducing different side chains, 6-hydroxybenzothiazole-2-carboxamide derivatives achieve precise regulation of MAO-B activity, which is uncommon among current medications. Furthermore, 6-hydroxybenzothiazole-2-carboxamide derivatives demonstrate favorable pharmacokinetic properties and bioavailability in both *in vitro* and *in vivo* experiments, providing strong support for their potential use as therapeutic agents for neurodegenerative diseases. The extreme toxicity and adverse effects of these clinical studies have imposed limitations. In order to tackle this issue more effectively, a range of derivatives of the novel compound 6-hydroxybenzothiazole-2-carboxamide have been discovered. These derivatives involve modifications to the amide substituent, resulting in several powerful compounds with various side chains. These compounds have demonstrated distinct effects in selectively inhibiting MAO-B, making them potential targeted therapeutic agents for neurodegenerative diseases.

Quantitative Structure-Activity Relationship (QSAR) is a scientific methodology that involves modeling and predicting the biological activity of a molecule based on quantitative connections between its structural characteristics (Cherkasov et al., 2014). Computer-aided drug design has evolved into a mature and promising field of study, focusing on quantitative structural connections (Santos-Filho and Hopfinger, 2001). The conventional approach to drug design is marked by inefficiency, lengthy processes, and exorbitant expenses. Consequently, there is a pressing need for novel research and development methodologies to enhance the efficiency and effectiveness of drug design (Duch et al., 2007). Computer-aided drug design, on the other hand, has been widely recognized and applied in drug discovery and development for its advantages of high efficiency, less time-consuming and low cost (Zheng et al., 2013). Computer-aided drug design has gained widespread recognition and use in the field of drug discovery and development due to its notable benefits of increased efficiency, reduced time requirements, and lower costs (Wang et al., 2021). The current investigation included the development of a 3D-QSAR model for novel derivatives of 6-hydroxybenzothiazole-2-carboxamides. This was achieved by the use of comparative molecular similarity indices analysis (COMSIA) and molecular docking investigations. This approach has significantly facilitated the comprehension of the correlation between the structure and activity of the compounds. Based on the findings of QSAR and molecular docking, we may enhance the activity of drugs by making further structural modifications.

Understanding the underlying mechanisms that govern the binding capabilities and stability of our compounds is crucial for the rational design of novel therapeutic agents. Therefore, in this study, we have not only presented the experimental results but also conducted a thorough mechanistic analysis to elucidate the factors contributing to the superior performance of the identified compounds.

## 2 Materials and methods

### 2.1 Experimental

QSAR is a scientific method for predicting the biological activity of molecules based on their structural features. Compared with traditional QSAR methods, the 3D-QSAR approach employed in this study offers significant advantages. Traditional QSAR methods primarily rely on two-dimensional molecular descriptors, neglecting conformational changes of molecules in three-dimensional space. In contrast, the 3D-QSAR method takes into account the specific conformations of molecules within the active site, thereby enabling more accurate predictions of molecular biological activity. Furthermore, the COMSIA method used in our study enhances the predictive accuracy and reliability of the model by comprehensively considering multiple molecular fields, including steric, electrostatic, hydrophobic, hydrogen bond donor, and hydrogen bond acceptor fields.

#### 2.1.1 Analytical dataset

This research presented the structures and IC50 values of 36 newly discovered compounds of 6-hydroxybenzothiazole-2-carboxamide (Table 1) (Al-Saad et al., 2024). To mitigate the asymmetry of the dataset, the IC50 values of all the substances were transformed to -log (IC50) + 6. Subsequently, the dataset was meticulously split into a training set consisting of 29 compounds and a test set consisting of 7 compounds using a random selection process. The training set was utilized to develop the 3D-QSAR model, while the test set was employed for model validation to ensure its predictive accuracy and robustness.

**TABLE 1:** The experimental and predicted data obtained from the COMSIA model.

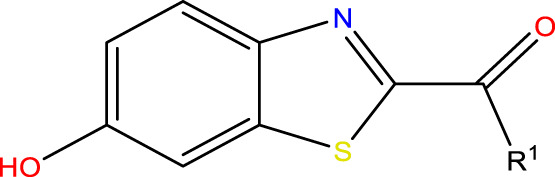					
Compound	R^1^	IC50 (μM)	Experimental	Predicted	Residual
1	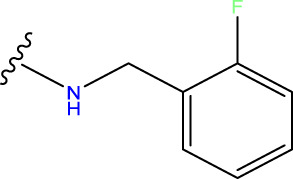	0.279 ± 0.036	6.554	6.865	−0.214
2	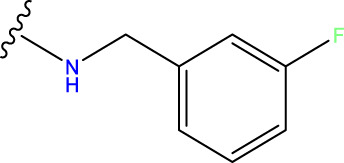	0.622 ± 0.043	6.206	6.651	−0.173
3	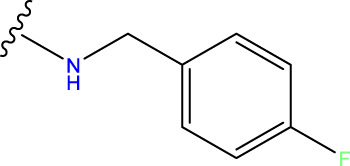	0.397 ± 0.051	6.401	6.484	−0.06
4	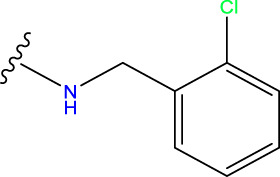	0.146 ± 0.018	6.836	6.788	0.011
5	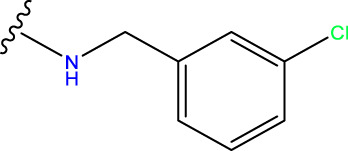	0.437 ± 0.053	6.360	6.682	−0.167
6	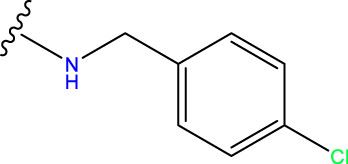	2.97 ± 0.50	5.527	6.342	−0.143
7	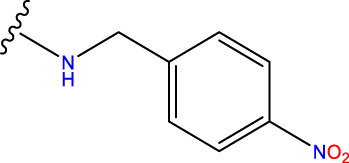	1.04 ± 0.02	5.983	5.982	−0.014
8	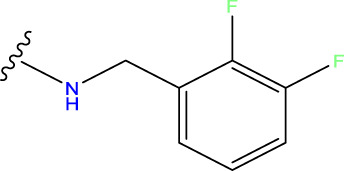	0.025 ± 0.001	7.602	7.249	0.237
9	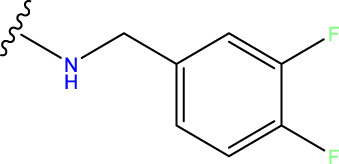	0.033 ± 0.003	7.482	6.856	0.159
10	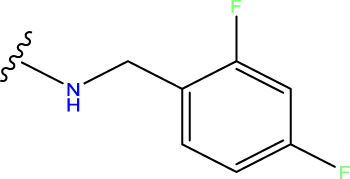	0.100 ± 0.011	7	7.056	−0.145
11	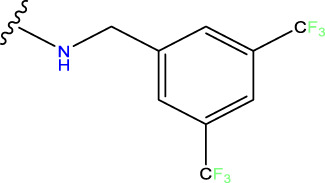	0.129 ± 0.012	6.889	6.838	−0.009
12	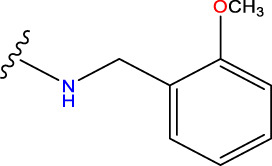	0.258 ± 0.088	6.588	6.376	0.101
13	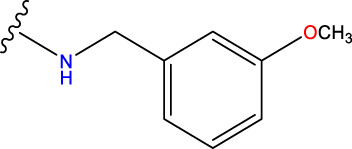	1.94 ± 0.38	5.712	5.791	−0.148
14	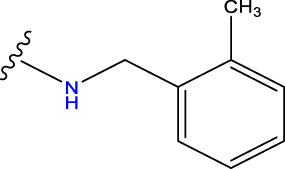	0.147 ± 0.035	6.833	6.373	0.17
15	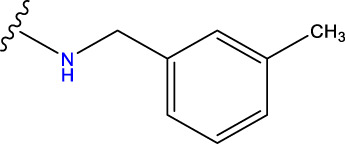	0.282 ± 0.080	6.550	6.177	0.156
16	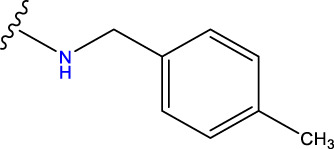	2.16 ± 0.14	5.666	6.03	−0.112
17	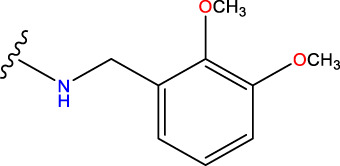	1.22 ± 0.03	5.914	5.89	−0.032
18	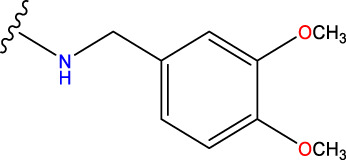	3.21 ± 0.55	5.494	5.33	0.061
19	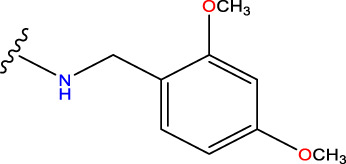	0.971 ± 0.107	6.013	5.966	0.007
20	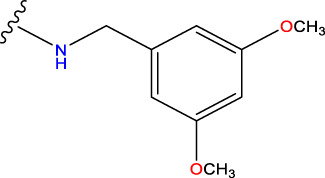	1.80 ± 0.10	5.745	5.675	−0.056
21	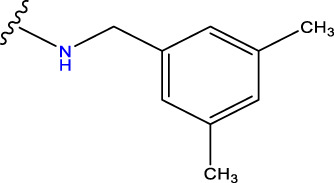	0.230 ± 0.027	6.638	6.293	0.014
22	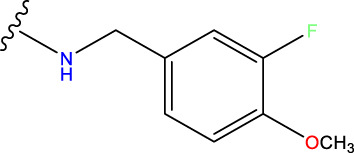	0.851 ± 0.123	6.070	6.163	−0.098
23	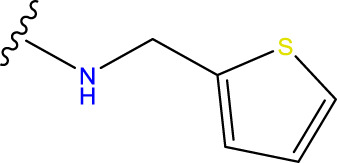	0.250 ± 0.001	6.602	6.364	0.119
24	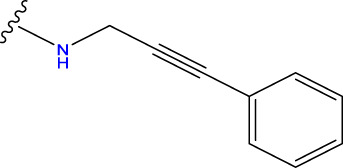	0.944 ± 0.116	6.025	6.14	−0.013
25^a^	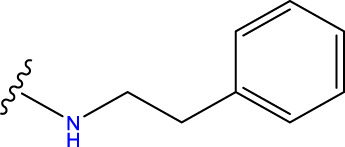	0.041 ± 0.005	6.5544	5.79	0.028
26^a^	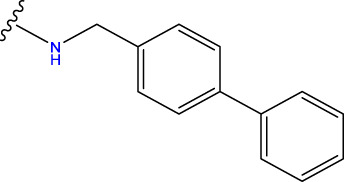	2.09 ± 0.18	6.2062	6.01	0.015
27	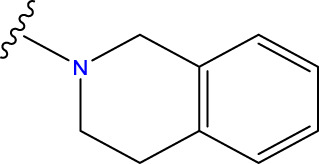	1.18 ± 0.01	7.3872	7.566	0.009
28^a^	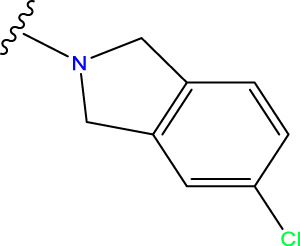	0.140 ± 0.015	6.401	6.624	0.04
29^a^	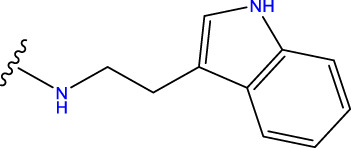	0.140 ± 0.016	6.836	6.571	−0.018
30^a^	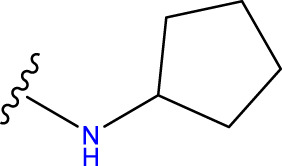	0.842 ± 0.067	6.360	5.796	0.099
31	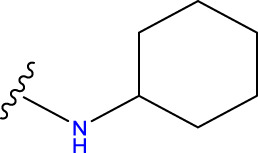	0.011 ± 0.005	5.680	5.783	−0.156
32	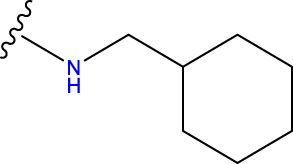	0.118 ± 0.005	5.928	5.998	0.109
33^a^	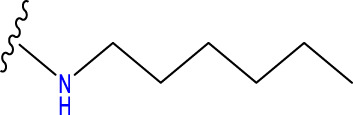	0.072 ± 0.008	5.983	6.18	0.046

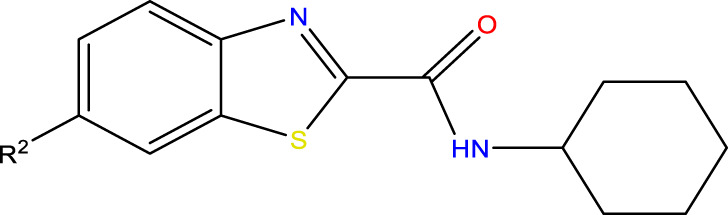					
Compound	R2	IC50 (μM)	Experimental	Predicted	Residual
34^a^	-OCH3	0.428 ± 0.019	7.602	6.862	−0.041
35	-H	3.08 ± 0.25	6.854	6.895	0.013

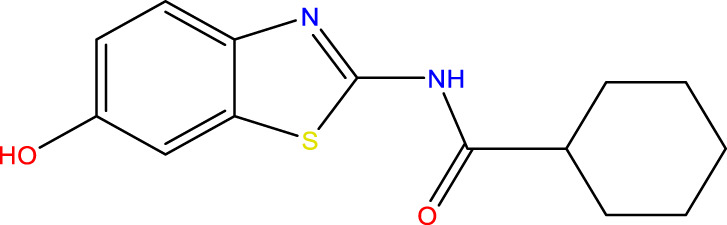					
Compound	—	IC50 (μM)	Experimental	Predicted	Residual
36	—	0.779 ± 0.018	6.854	6.791	0.013

^a^ The test set data.

#### 2.1.2 Structural optimization

The construction of all compounds was carried out using ChemDraw software. The designed compounds were then put into Sybyl-X software for spatial structure optimization. The Sybyl-X program used the Tripos force field and Powell gradient methods, which are the default settings of the system, to reduce the energies of all the compounds. The resulting minimized structures were then employed as the starting conformations for the COMSIA approach (Yu et al., 2015).

#### 2.1.3 Compound superposition

Structural alignment of compounds is a critical step in 3D-QSAR analysis, which directly affects the subsequent prediction of drug activity (Patel et al., 2008). Compound alignment can be divided into two approaches: one centered on ligand (ligand)-based common structures and the other on receptor (receptor)-based small molecule targets. In this study, we used the former approach, which centers on the common structure of the most active compound 31, to align all compounds to ensure consistency and accuracy of the QSAR analysis.

**FIGURE 1:**
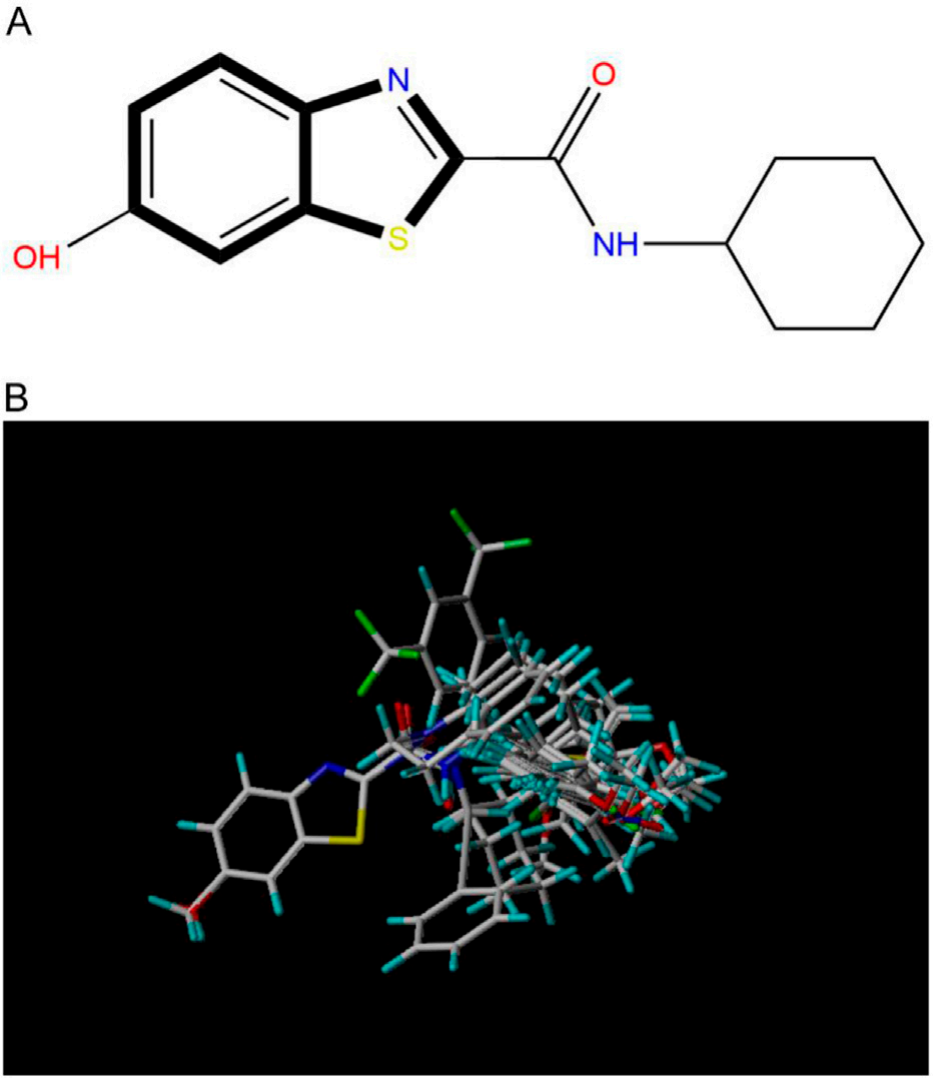
Compound 31 used as a reference to evaluate all the compounds in the collection. **(A)** The bold wording signifies the universal structure shared by all compounds. **(B)** All compounds are organized based on a shared structure.

Compound overlay is critical in 3D-QSAR analysis, ensuring that all compounds involved in the modeling are spatially comparable. Since QSAR modeling relies on a quantitative relationship between a compound’s structure and its biological activity, it is important to ensure that these compounds are structurally correctly aligned and compared. By overlaying the most active compounds (e.g., compound 31 in this study) as a benchmark (Figure 1), we are able to more accurately identify which structural features contribute to biological activity, which can guide subsequent drug design and optimization.

#### 2.1.4 Study conducted by COMSIA

In order to conduct COMSIA investigations, a grid was created with a side length of 0.2A in all places where the molecules were constructed. Additionally, a 4A border was used to identify all sections of the stacked molecules. The steric, electrostatic, hydrophobic, and hydrogen bonding fields, which include hydrogen bond acceptors and hydrogen bond donors, were computed for each grid point using the default probes given with COMSIA. A quantitative correlation model between molecular field characteristics and affinity was created using partial least squares (PLS) after acquiring the molecular field at each grid point. The statistical significance of the model was further assessed using Leave-one-out and cross validation techniques, and the optimal number of main components for the model was identified. A 3D-QSAR model was constructed by using several main components that were selected using the best interaction check results. The model was built without interaction checks and then used to predict the affinity of the compounds in the test set (Li et al., 2012a; Li et al., 2012b).

#### 2.1.5 Verification of the 3D-QSAR model

Generally, greater values of q^2^, R^2^, and F, together with lower values of SEE, indicate a good capacity to fit the data. Nevertheless, relying just on these statistical metrics does not completely showcase the model’s predictive capacity. Additional validation is necessary to thoroughly assess the model’s dependability, resilience, and predictive power (Yu et al., 2015). The COMSIA model was validated using Leave-one-out cross-validation and the optimal number of components was determined. The q^2^ value, calculated as the cross-validation coefficient, yielded a value of 0.569, indicating good predictive power of the model. Similarly, the r^2^ value, which measures the goodness of fit between the model predictions and the experimental data, was found to be 0.915, demonstrating a high degree of fit. These statistical findings, along with the lower standard error of estimate (SEE = 0.109) and higher F-value (52.714), confirm the adequacy of the COMSIA model.

#### 2.1.6 Verification of the 3D-QSAR model

Generally, greater values of q^2^, R^2^, and F, together with lower values of SEE, indicate a good capacity to fit the data. However, relying solely on these statistical metrics does not fully demonstrate the model’s predictive capacity and reliability. Therefore, multiple evaluation metrics have been employed to thoroughly assess the model.

The 3D-QSAR model was developed using the training set, which consisted of 29 compounds randomly selected from the entire dataset. The remaining 7 compounds were used as the test set to validate the model. The predictive performance of the model was evaluated using both internal and external validation techniques.

##### 2.1.6.1 Internal validation

Cross-validation coefficient (q^2^): This metric was used to assess the model’s ability to predict the activities of compounds within the training set. A q^2^ value of 0.569 was obtained, indicating good predictive power of the model.

Coefficient of determination (r^2^): This metric measures the degree of fit between the model predictions and the experimental data for the training set. An r^2^ value of 0.915 was achieved, demonstrating a high degree of correlation between the predicted and experimental activities.

Standard error of estimate (SEE): This metric quantifies the average deviation of the predicted values from the experimental values for the training set. A low SEE value of 0.109 was obtained, indicating the model’s accuracy in predicting compound activities.

F-value: This metric provides a measure of the statistical significance of the model. A high F-value of 52.714 was obtained, further confirming the adequacy of the model.

##### 2.1.6.2 External validation

To further evaluate the predictive ability and reliability of the CoMSIA model, external validation was performed. External validation parameters, including the number of compounds in the test set (ntest), the mean value of compound activity in the training set (tr), the correlation coefficient between experimental and predicted activity of compounds in the test set (R^2^_ext_), and the adjusted correlation coefficient (R^2^_m_), were calculated. Typically, a value of R^2^_ext_ greater than 0.5 is considered indicative of a statistically reliable model with strong predictive capability. The model exhibited R^2^_ext_ and R^2^_m_ values of 0.688 and 0.642, respectively, clearly demonstrating its robustness and predictive ability.

Therefore, we conducted external validation using the test set. The following metrics were calculated:

There are two often used external validation techniques that utilize the following formulas:

$$R_{\mathrm{e}xt}^{2}=1-\frac{{\sum_{i=1}^{ntest}\left(y_{i}-{\tilde{y}}_{i}\right)}^{2}}{\sum_{i=1}^{ntest}{\left(y_{i}-{\bar{y}}_{tr}\right)}^{2}}$$

The equation involves the variables ntest, $\bar{y}$ _tr_, y _*i*_ and $\tilde{y}$ _*i*_. Ntest represents the number of compounds in the test set, $\bar{y}$ _tr_ represents the average value of compound activity in the training set, and y _*i*_ and $\tilde{y}$ _*i*_ represent the experimental and anticipated values of compound activity in the test set, respectively. Typically, a value of R^2^_ext_ greater than 0.5 is seen as indicative of a statistically reliable model with strong predictive capability (Mouchlis et al., 2012).

Furthermore, the parameter R^2^_m_ may be further validated to assess the appropriateness of the model using the following equation:

$$R_{m\left(overall\right)}^{2}=R^{2}*\left(1-\sqrt{R^{2}-R_{0}^{2}}\right)$$

R^2^ in this equation denotes the squared value of the correlation coefficient, which measures the degree of correlation between the anticipated and experimental activity levels for all substances. Conversely, R^2^_0_ is the squared correlation coefficient between predicted and experimental values when the intercept is adjusted to 0.

R^2^_ext_: This metric measures the correlation between the predicted and experimental activities for the test set. A value of R^2^_ext_ > 0.5 is generally considered indicative of a statistically reliable model. In this study, an R^2^_ext_ value of 0.688 was obtained, demonstrating the model’s predictive power on an independent dataset.

R^2^_m_: This metric is an adjusted correlation coefficient that takes into account the number of compounds in the test set. A value of R^2^_m_ > 0.5 further confirms the model’s appropriateness. An R^2^_m_ value of 0.642 was obtained, indicating the model’s robustness.

In summary, the 3D-QSAR model developed in this study has been thoroughly validated using multiple evaluation metrics, including q^2^, r^2^, SEE, F-value, R^2^_ext_, and R^2^_m_. These metrics collectively demonstrate the model’s reliability and predictive power, providing confidence in its application for the development of novel MAO-B inhibitors.

#### 2.1.7 Molecular docking

Molecular docking is a computational technique that simulates the interaction between a small molecule ligand and a biological macromolecule receptor. Compared with traditional molecular docking methods, the flexible docking approach employed in this study better accounts for conformational changes of both the ligand and the receptor during the binding process. By allowing a certain degree of conformational adjustment for the ligand and receptor during docking, the flexible docking method can more accurately simulate the real biomolecular interaction process. Additionally, the Sybyl-X software platform used in our study provides powerful tools for molecular mechanics optimization and energy evaluation, further enhancing the accuracy and reliability of the docking results.

#### 2.1.8 Molecular dynamics study

Molecular dynamics (MD) simulations of the key screened protein-ligand complex were conducted using the GROMACS 2023.2 package and the CHARMM 36 force field to examine conformational changes and dynamic behavior at the atomistic level (Guo and Liu, 2024; Tak et al., 2024). The ligand topology of the compound was generated via the CGenFF web server, and Na+/Cl-counter ions were added to neutralize the complex structures (Fischer et al., 2015). The CHARMM-modified TIP3P water model was selected, and the termini were designated as “NH3+” and “COO-” due to the N-terminal residue being methionine (MET), necessitating this interactive selection to avoid pdb2gmx choosing an incompatible terminus type intended for carbohydrates. Protein-specific termini were therefore chosen.

The system’s energy was minimized initially using the steepest-descent algorithm followed by the conjugate gradient method (Donnelly et al., 2021). Long-range electrostatic interactions were computed using the Particle Mesh Ewald (PME) method, while the LINear Constraint Solver (LINC) algorithm was employed to calculate Lennard-Jones and Coulomb interactions with a 10Å cutoff distance (Hess, 2008). Temperature coupling was maintained at 300K using the Berendsen thermostat (V-rescale) coupling algorithm, and pressure coupling was maintained at 1 bar using the Parrinello–Rahman pressure coupling algorithm (Karwasra et al., 2020). Post-energy minimization, the system was equilibrated for 100 ps each under NVT and NPT conditions to stabilize volume, pressure, and temperature. The system then underwent a 50 ns MD production run with coordinates saved at consistent intervals of 2 fs (Metwally and Hathout, 2015).

From the final trajectory data, analyses including root mean square deviation (RMSD) (Maruyama et al., 2023), root mean square fluctuation (RMSF), and hydrogen bond (H-bond) analysis were performed using GROMACS modules. The global motion of all complexes was further analyzed with a free energy landscape (FEL) study, as detailed in previous research (Abdelsattar et al., 2021). Binding free energy calculations were performed using the molecular mechanics/Poisson-Boltzmann surface area (MM-PBSA) method, a computational technique used to estimate binding free energies of biomolecular complexes lilce protein-ligand interactions (Xue et al., 2019). This method integrates molecular mechanics force fields, continuum solvent models,and empirical solvation energy terms to estimate the thermodynamic properties of protein-ligand interactions. MM-PBSA allows for the decomposition of binding free energy into various components such as van der Waals interactions, electrostatic interactions, solvation-free energy,and entropy contributions. This decomposition aids in understanding individual energetic contributions, which can inform specific aspects of ligand optimization.

MM-PBSA results can be compared with experimental binding affinities to validate the accuracy of computational predictions, an essential step in building confidence in the computational approach and its applicability in drug discovery. The binding-free energy of the selected compound was calculated conceming the protease protein from the last 50 ns of the MD trajectory using the gmx_MMPBSA tool (Tan et al., 2024; Valdés-Tresanco et al., 2021).

## 3 Results

### 3.1 Statistical findings derived from the COMSIA model

**FIGURE 2:**
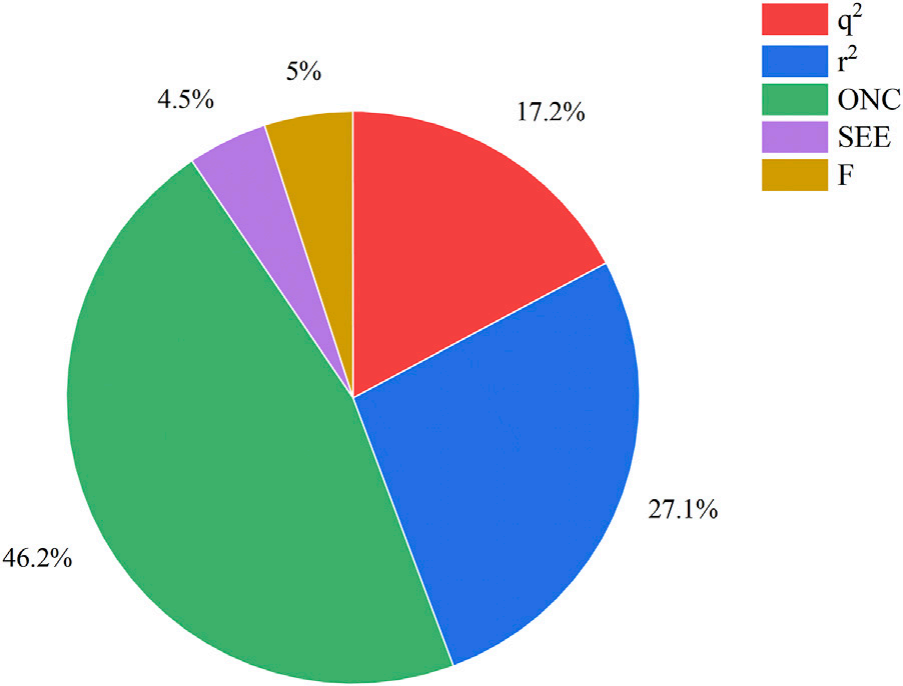
The force field contribution of the most optimal COMSIA model.

The COMSIA approach generates five distinct molecular force fields with various levels of contribution. The proportions of these five force fields are as follows: spatial force (17.2%), electrical force (27.1%), hydrodynamic force (46.2%), hydrogen bonds donor (4.5%), and the hydrogen bond acceptor (5.0%). By organizing and merging all five of these molecules fields of force, a total of 10 sets of model were created. Upon careful analysis of these 10 sets of models, it was concluded that the ESH model exhibited the highest level of satisfaction, as shown by the findings presented in Table 2. Figure 2 illustrates the effect of this model. The most efficient COMSIA model yielded a q2 statistic of 0.569, a superior r2 value of 0.915, an optimal group score of 3, a reduced ESHAD of 0.109, and an elevated F value of 52.714.

**TABLE 2:** Statistical parameters of the COMSIA model.

No.	Model	q^2^	ONC	r^2^	F value	SEE	r^2^_pre_
1	ESHAD	0.569	3	0.915	52.714	0.109	5.783
2	ESA	0.537	4	0.861	53.130	0.167	5.736
3	EHD	0.369	5	0.921	44.644	0.101	5.737
4	ESD	0.466	4	0.856	52.597	0.172	5.605
5	ESHA	0.551	3	0.915	52.720	0.109	5.654
6	ESHD	0.528	3	0.913	52.129	0.112	5.681
7	EHAD	0.339	4	0.922	54.834	0.101	5.729
8	SHAD	0.552	4	0.780	37.428	0.236	5.722
9	ESAD	0.499	5	0.857	42.624	0.171	5.601
10	ESH	0.516	4	0.912	51.785	0.113	5.658

S, steric; E, electrostatic; H, hydrophobic; D, hydrogen bond donor; A, hydrogen bond acceptor.

### 3.2 Results of COMSIA model validation

The COMSIA model was further validated through external validation techniques. The cross-validation coefficient (q^2^) and the coefficient of determination (r^2^) were calculated to assess the model’s predictive performance. The q^2^ value, given by the formula:

$$q^{2}=1-\frac{{\sum_{i-1}^{n}\left(y_{i}-{\tilde{y}}_{i}\right)}^{2}}{{\sum_{i-1}^{n}\left(y_{i}-\bar{y}\right)}^{2}}$$

yielded a value of 0.569, indicating good predictive power of the model. Similarly, the r^2^ value, calculated using the formula:

$$r^{2}=1-\frac{{\sum_{i-1}^{n}\left(y_{i}-{\tilde{y}}_{i}\right)}^{2}}{{\sum_{i-1}^{n}\left(y_{i}-\bar{y}\right)}^{2}}$$

was found to be 0.915, demonstrating a high degree of fit between the model predictions and the experimental data. These statistical findings, along with the lower standard error of estimate (SEE = 0.109) and higher F-value (52.714), confirm the adequacy of the COMSIA model.

**FIGURE 3:**
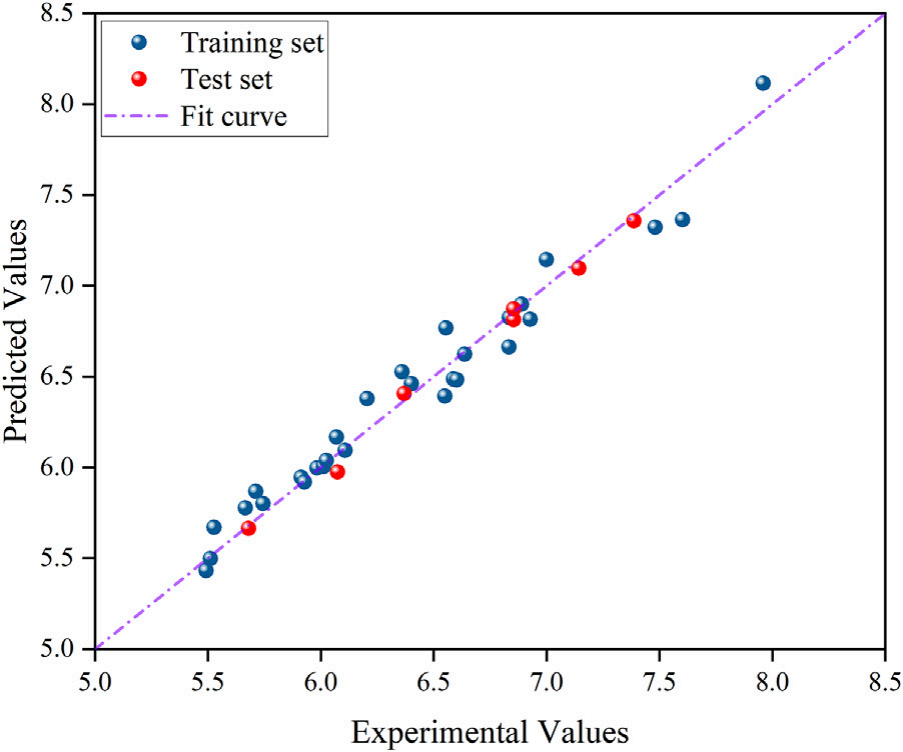
Plot comparing the predicted and experimental values of COMSIA model compounds.

The experimental values of the model in Figure 3 exhibit a strong linear correlation with the anticipated values.

### 3.3 Summary of external validation parameters

In order to fully assess the predictive ability and reliability of the CoMSIA model, we performed external validation and summarized the key validation parameters. Table 3 lists the external validation parameters of the model, including the number of compounds in the test set (ntest), the mean value of compound activity in the training set (tr), the correlation coefficient between experimental and predicted activity of compounds in the test set (R2ext), and the adjusted correlation coefficient (R2m). Together, these parameters demonstrate the robustness and predictive ability of the constructed model.

**TABLE 3:** Summary of external validation parameters for the CoMSIA model.

Parameters	ntest	tr	R^2^_ext_	R^2^_m_
Value	7	6.502	0.688	0.642

Note: Both R^2^_ext_ > 0.5 and R^2^_m_ > 0.5 indicate that the model has statistically reliable predictive power.

### 3.4 Contour maps generated with COMSIA

**FIGURE 4:**
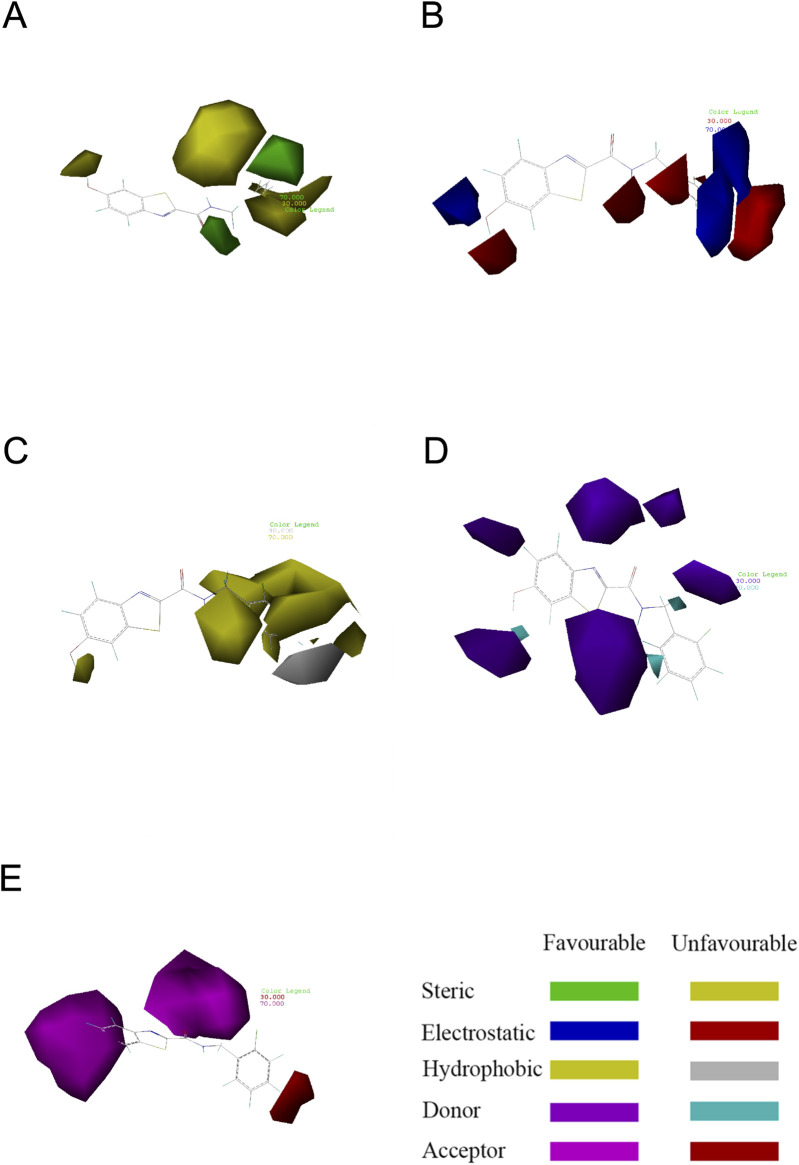
Contour map showing the optimal configuration of compound 31. **(A)** In the steric field, the color green indicates a favorable situation, whereas yellow indicates an unfavorable one. **(B)** In an electrostatic field, the color blue indicates a positive field, whereas the color red indicates a negative field. **(C)** In a hydrophobic environment, the color yellow indicates a favorable condition, whereas white indicates an unfavorable one. **(D)** Purple represents advantageous hydrogen bond donor fields, whereas cyan represents unfavourable hydrogen bond donor fields. **(E)** Favorable (magenta) and unfavorable (red) hydrogen bond acceptor regions.

The contour plots of the COMSIA model provide insight into the precise correlation between the compound’s structure and its pharmacological action (Figure 4) (Xiao et al., 2021). The experiment included creating several contour plots of molecular steric field, electrostatic field, hydrophobic field, hydrogen bond donor field, and hydrogen bond acceptor field using the StDev*Coeff technique. The relationship between compound structure and pharmacological action was discerned by the observation of color variations in various areas of the contour plots (Liu et al., 2018).

Compound 31 was chosen as the reference structure in the contour plot of the CoMSIA model seen in Figure 4. (Figure 4A) displays a green contour in the vicinity of the R^1^ location, showing a favorable spatial region where the presence of a large group substituent improves the biological activity. Furthermore, the presence of the yellow contour next to the R^1^ location suggests that even minor group substitutions might have an impact on the activity. Referring to (Figure 4B), the presence of a blue contour in the vicinity of the R^1^ location indicates the presence of an electrostatic field. Hence, the inclusion of cationic substituents at this location would enhance the biological efficacy. The hydrophobic contour is shown by the yellow contour around the point of R^1^ in (Figure 4C). This suggests that the presence of a hydrophobic group at this location enhances the action. The presence of the white contour line signifies that the hydrophilic group strongly promotes the biological activity. The presence of a hydrophilic group at this location enhances the action. Compound 31, with a moiety in the R^1^ position, had much superior activity compared to the other compounds. Both (Figure 4D) and (Figure 4E) show contours at the R^1^ position that suggest hydrogen bonding. These contours provide direction for the incorporation or elimination of hydrogen bond donor or acceptor groups. The contour maps depicting hydrogen bond acceptors are shown with fuchsia and red contours. Introducing hydrogen bond acceptor groups around the fuchsia outlines enhances activity, but the red contours indicate regions where hydrogen bond acceptor groups are unnecessary. The hydrogen bond donor contour map is shown using purple and blue-green contours. The incorporation of hydrogen bond donor groups in close proximity to the purple contour is advantageous for enhancing biological activity, whereas the occurrence of hydrogen bond donor groups near the blue-green contour is not anticipated.

### 3.5 Developed novel compounds and forecasted IC50 values using the 3D-QSAR model

The neuroprotective efficacy of the targeted medicine in MAO-B neurodegenerative illness was determined based on the primary parameters impacting it. Structural alteration of the reference molecule31 was conducted by following the contour lines and combining distinct molecular fields in the contour plot. Upon consolidating the contour findings, we designated the locations to be altered as R^1^ and R^2^. Due to the fact that hydrophobic fields increase the efficacy of the medicine, we used hydrophobic groups for structural modification. Hydrophobic groups include several types of compound groups, such as alkyl groups (including -C_n_H_2n_ +1, -CH = CH_2_, C_6_H_5_, etc.), halogen atoms (-X), and nitro (-NO_2_), among others. Utilizing these modification principles, we have successfully produced over 100 novel compounds. A total of over 100 compounds were optimized using SYBYL 2.1.1, and their pIC50 values and overall scores were predicted. The top-performing compounds, namely, compounds 31. a-31.j3, were chosen for further investigations. Table 4 displays the molecular structures of these 10 compounds together with their estimated pIC50 values and overall scores. The data clearly indicates that compound 31.j3 exhibits the greatest pharmacological activity value, suggesting that it has significant potential for neuroprotective action.

**TABLE 4:** New compounds designed and their predicted values and total docking scores.

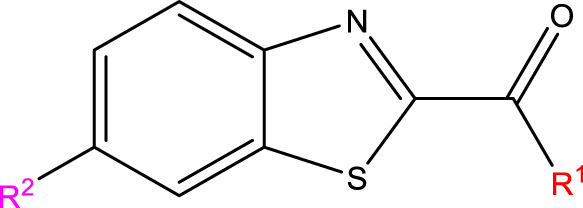				
No.	R^1^	R^2^	pIC50 predicted by CoMSIA	Total scores
31	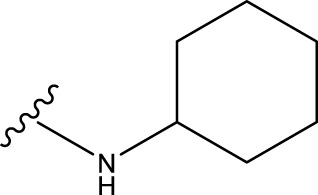	-OH	5.783	6.0648
31.a	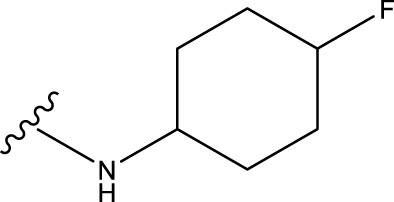	-OH	6.431	5.3026
31.b	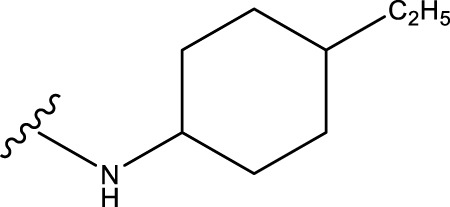	-OH	6.386	6.0734
31.c	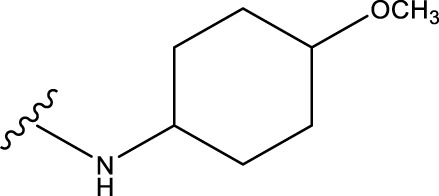	-OH	6.375	6.3952
31.d	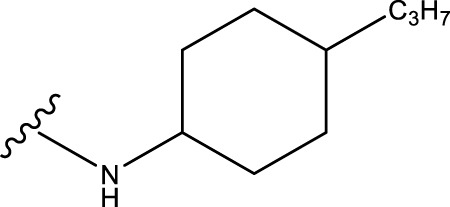	-OH	6.359	7.2428
31.e	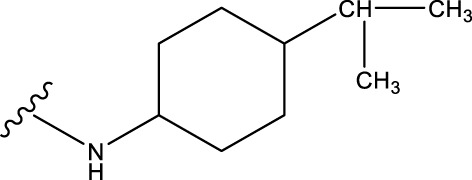	-OH	6.452	7.8233
31.f	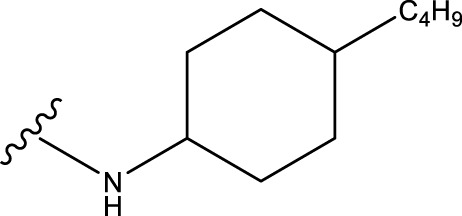	-OH	6.355	6.2652
31.g	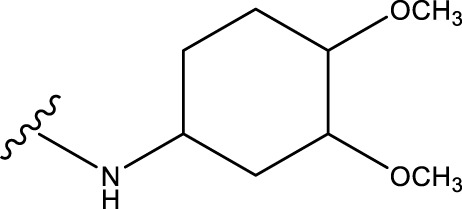	-OH	6.247	6.4829
31.h	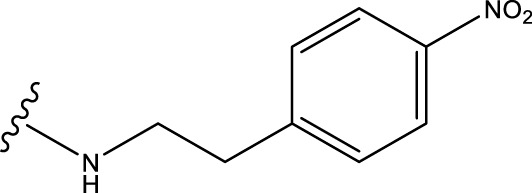	-OCH3	5.739	8.0273
31.i	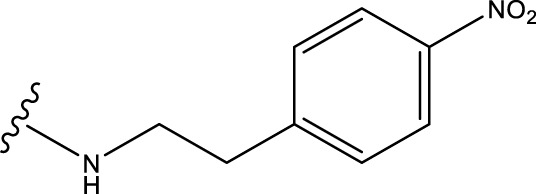	-H	6.464	7.0429
31.j3	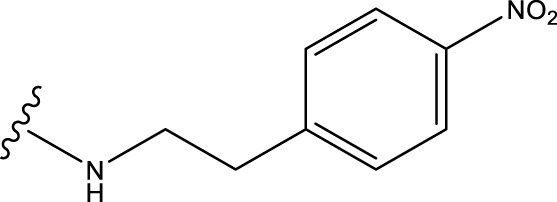	-OH	6.391	8.1522

### 3.6 Molecular docking experiments

Before preparing for molecular docking, we performed the necessary pre-processing of the MAO-B receptor fragment. First, the crystalline water molecules in the receptor structure were removed, as these water molecules are not present under physiological conditions and may interfere with the proper binding of the ligand to the receptor. Second, missing hydrogen atoms were hydrogenated to restore the integrity and accuracy of the receptor structure. These pre-processing steps are essential to ensure the accuracy and reliability of molecular docking results.

**FIGURE 5:**
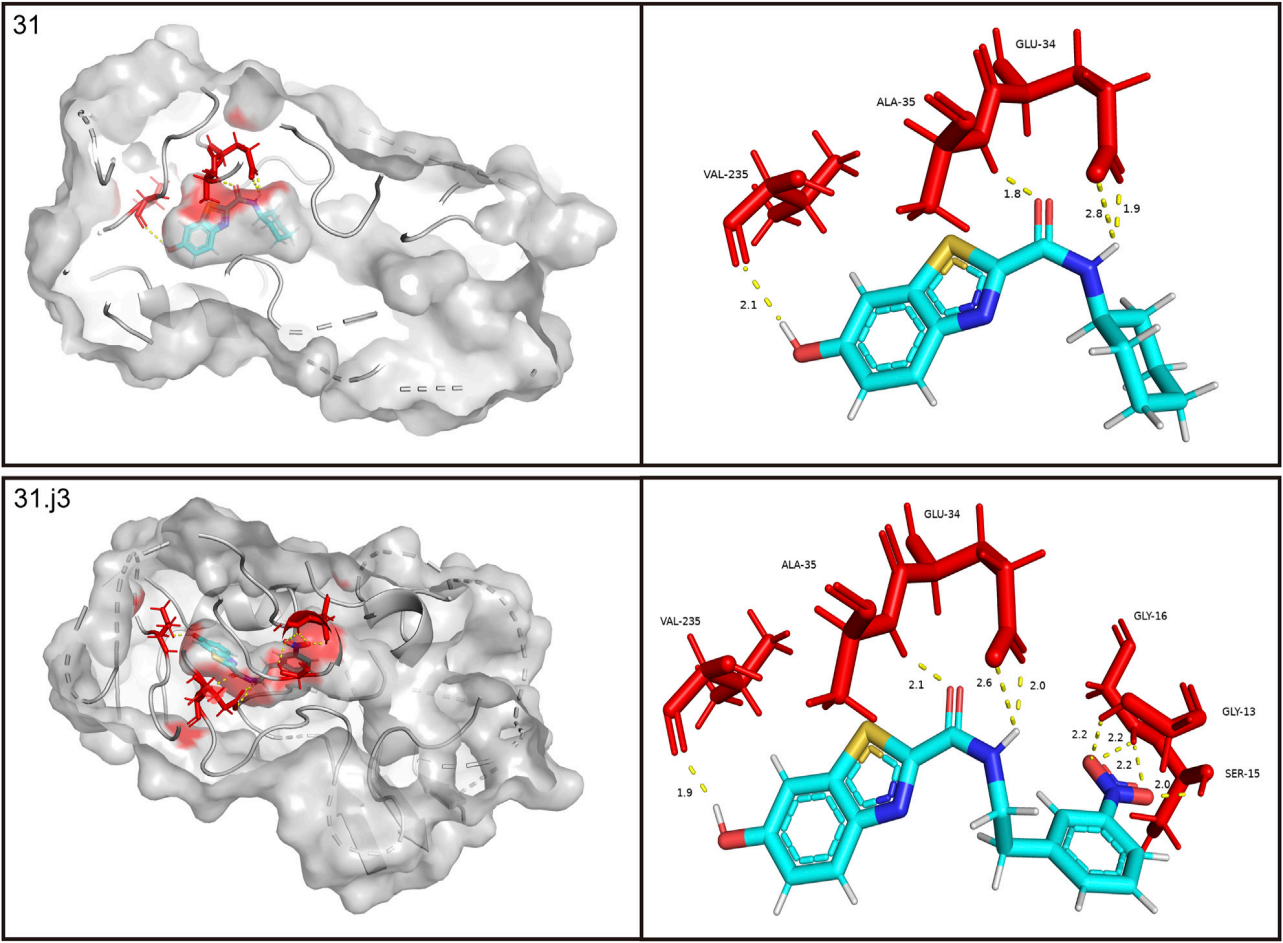
Performing molecular docking of small molecule compounds 31 and 31.j3 with MAO-B target molecules that are closely related (PDB ID: 3PO7).

Drug molecules were loaded into the Sybyl-X programme and the molecular mechanics of the ligand small molecules were improved using the conjugate gradient method. The optimisation procedure was carried out using the Tripos force field with the energy convergence criterion set at 0.01 kcal/(mol-A) and a maximum iteration limit of 106. After molecular mechanics optimisation, the best active conformation was selected for molecular docking studies. To prepare for subsequent molecular docking, a small fragment of the MAO-B receptor (crystal structure of MAO-B obtained from the RCSB PDB Protein Data Bank, PDB ID: 3PO7) (Al-Saad et al., 2024) was removed from the crystalline water molecules and hydrogenated atoms. The original ligand in the MAO-B fragment was then extracted and its binding site was determined as shown in Figure 5.

The Sybyl-X program was used to perform flexible docking between small molecule ligands and receptors. Activity pockets were produced by identifying and using the binding sites of the target ligands. The threshold parameter was configured to a value of 0.5, the expansion factor was set to 1, and the docking process was executed using the Sybyl-Dock standard mode. The molecular conformational changes were preserved for a duration of 20 units of time. The evaluation of the interaction between the small molecule and the target was conducted using the total score function of the Sybyl-Dock module. The Total-Score function considers the influences of polarity, hydrophobicity, and enthalpy. A greater number indicates a stronger interaction between the medication molecule and the protein crystal.

Based on Figure 1 and COMSIA contour plots, it is evident that the primary parameters influencing the activity of 6-hydroxybenzothiazole-2-carboxamide derivatives are hydrophobicity and hydrogen bonding (as shown in Table 5). Consequently, the development of novel compound should be derived from this. Nevertheless, the docking test findings revealed that a portion of the 100 recently developed compounds had either low activity levels or were incapable of successfully completing docking with the tiny MAO-B molecules in space, as indicated by their extremely low docking scores. Through careful analysis of these matters, it was discovered that the creation of novel compounds is conducted at the 2D-QSAR. Hence, when compounds undergo 3D-QSAR, there is a potential for misalignment with tiny MAO-B molecules, resulting in diminished activity levels and docking scores. Compound 31.j3 is an archetypal illustration. According to Figure 5, compound 31.j3 may fold spatially to create a ring-shaped structure. This structure can effectively attach to the target pocket of the MAO-B fragment. Additionally, compound 31.j3 can make hydrogen bonds with three specific residues of the MAO-B fragment: VAL-235, ALA-35, GLU-34, GLY-16, GLY-13, and SER-15. Unlike other compounds, many are shown to be linked to less than five or even zero amino acid residues. This aligns with the discovery that compound 31.j3 had the greatest docking prediction score in Table 3. Compound 31.j3 is considered a potential contender due to its potential to defend against damage to the nervous system in neurodegenerative illnesses.

**TABLE 5:** Molecular docking interactions of potential compounds.

Name	Define	Role in bonding
Hydrogen Bonds	Hydrogen bonding is an interaction between a hydrogen atom (usually attached to a more electronegative atom such as nitrogen, oxygen, or fluorine) and another electronegative atom	Hydrogen bonds are essential for specificity and stability between ligands and receptors. By analyzing the number and strength of hydrogen bonds, it can help to understand the affinity and specificity of a compound
Hydrophobic Interactions	Hydrophobic interactions occur between the nonpolar amino acid side chains of a protein and the hydrophobic regions of a ligand	These interactions help to increase the binding energy and stabilize the presence of the ligand within the binding site
Ionic Interactions	Ionic interactions, also known as electrostatic interactions, occur between positively charged groups (e.g., lysine, arginine) and negatively charged groups (e.g., aspartic acid, glutamic acid)	This interaction is usually very strong and significantly enhances the stability of the ligand-protein complex
Van der Waals Forces	These are weak interactions that occur between atoms and occur when atoms are close enough together	Although individual van der Waals forces are weak, their cumulative effect during binding may significantly affect ligand stability

Based on Figure 1 and COMSIA contour plots, it is evident that the primary parameters influencing the activity of 6-hydroxybenzothiazole-2-carboxamide derivatives are hydrophobicity and hydrogen bonding. Consequently, the development of novel compounds should focus on these interactions. Indeed, compound 31.j3 forms hydrogen bonds with several key amino acid residues of the MAO-B receptor, as detailed in Table 6. Additionally, it exhibits favorable hydrophobic interactions with other residues. These interactions collectively contribute to the high docking score and binding affinity of compound 31.j3, making it a promising candidate for further development.

**TABLE 6:** The most important interactions in molecular docking.

Compound	Receptor residues	Type of interaction	Distance (Å)
31.j3	VAL-235	Hydrogen bond	2.8
31.j3	ALA-35	Hydrogen bond	3.0
31.j3	GLU-34	Hydrogen bond	2.7
31.j3	GLY-16	Hydrophobicity	3.5
31.j3	GLY-13	Hydrophobicity	3.2
31.j3	SER-15	Hydrophobicity	3.9

### 3.7 Molecular dynamics simulation

The RMSD rapidly increases from 0 to around 1.0 Å within the first 2000 frames, indicating initial adjustments as the system equilibrates. Between 2000 and 6,000 frames, the RMSD fluctuates between 1.0 Å and 1.5 Å, suggesting that the complex is exploring conformational space but remains relatively stable around these values. The red trend line indicates a slight upward trend, which might suggest a gradual conformational change or adjustment in the complex over time. The RMSD continues to fluctuate but shows a slight increase toward the end of the simulation, reaching up to 2.0 Å. Despite the fluctuations, the RMSD values do not deviate significantly, indicating that the complex does not undergo major structural changes and remains relatively stable throughout the simulation.

**FIGURE 6:**
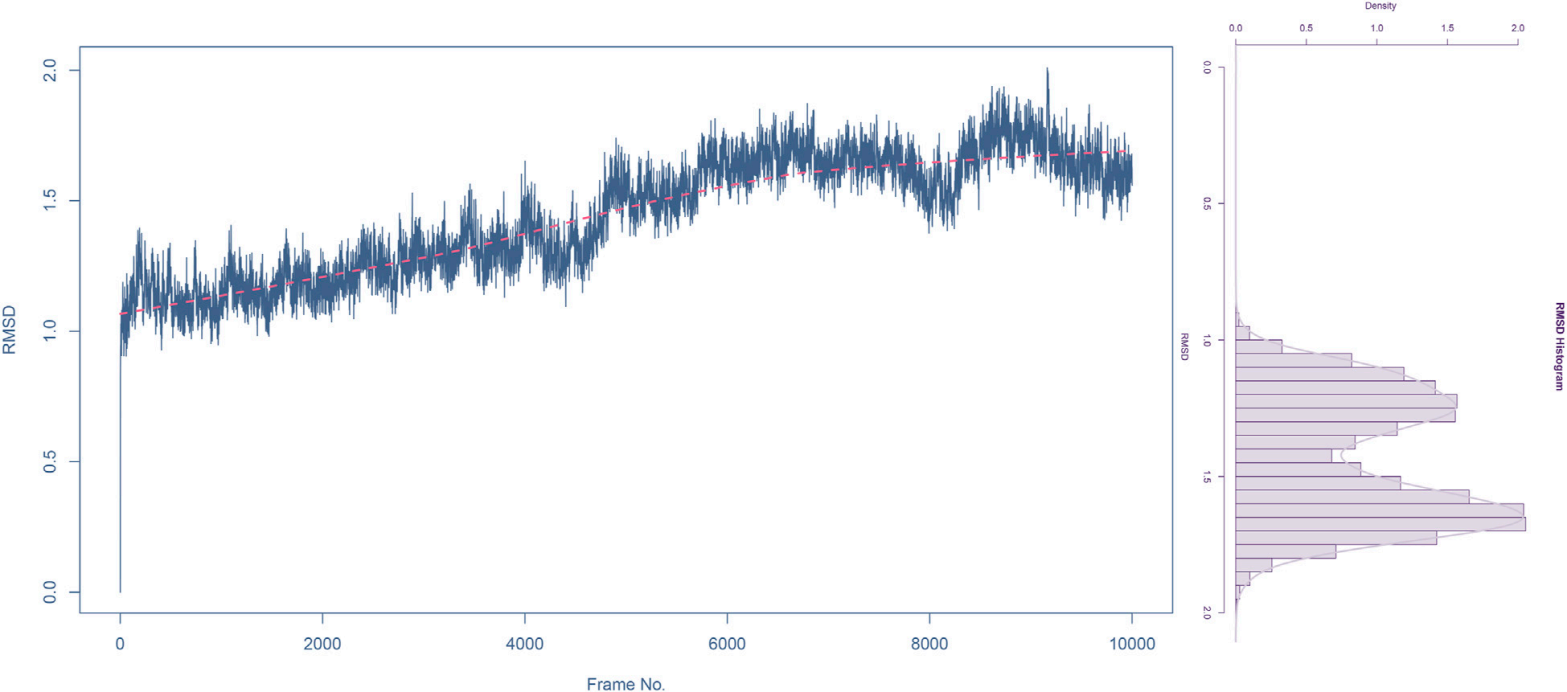
Root mean square deviation (RMSD) analysis of compound 31.j3 in complex with target protein (PDB ID: 3PO7) During molecular dynamics simulation.

The histogram shows the frequency distribution of RMSD values throughout the simulation. Most RMSD values lie between 1.0 Å and 2.0 Å, with the highest density around 1.5 Å (Figure 6). This indicates that the complex remains predominantly stable within this range. The peak around 1.5 Å suggests this is the most probable conformation of the complex during the simulation, reflecting its equilibrium state.

The RMSD analysis indicates that the complex of compound 31.j3 with the target protein (PDB ID: 3PO7) achieves stability after an initial equilibration phase. The RMSD values fluctuating around 1.5 Å with no significant deviations suggest that the binding of compound 31.j3 is stable over the simulation period. The slight upward trend in the RMSD trend line might warrant further investigation, but overall, the complex maintains a stable conformation, making compound 31.j3 a promising candidate for further experimental validation and development.

**FIGURE 7:**
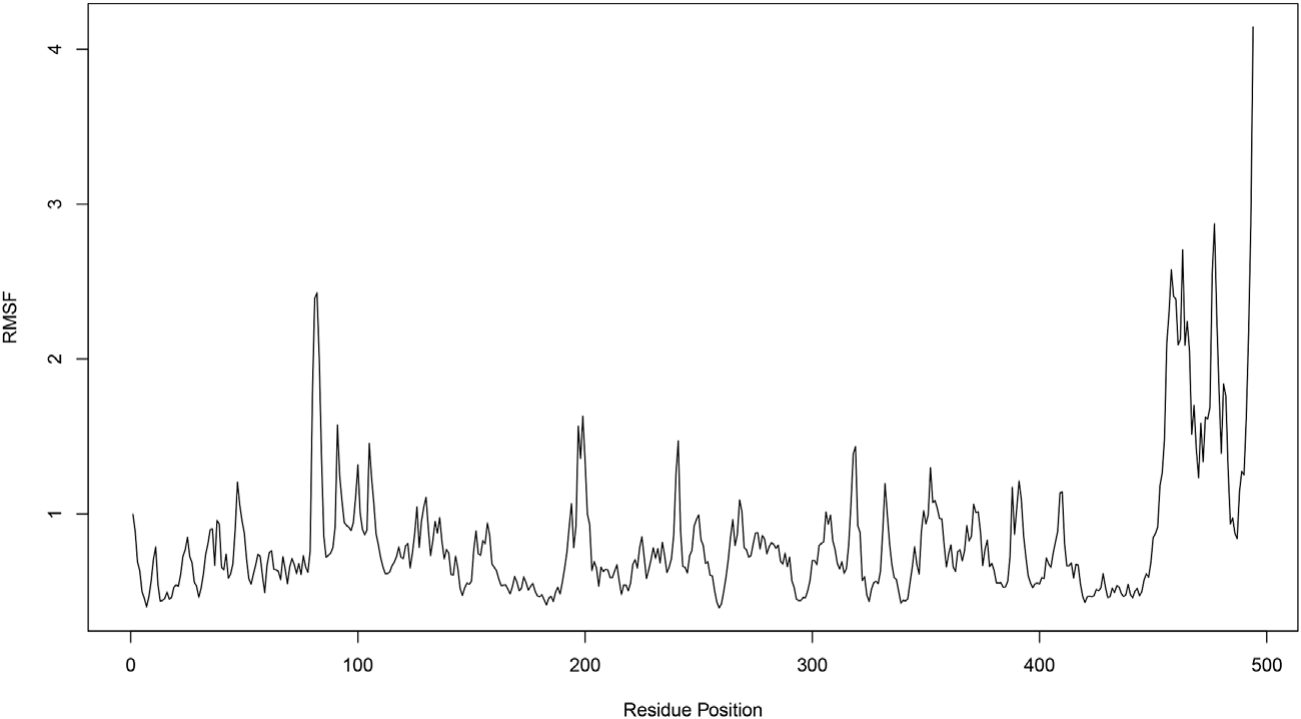
Root mean square fluctuation (RMSF) analysis of compound 31.j3 in complex with target protein (PDB ID: 3PO7).

The Root Mean Square Fluctuation (RMSF) plot provides insight into the flexibility of each residue in the protein throughout the molecular dynamics simulation. Higher RMSF values indicate greater flexibility, while lower values suggest more rigid regions. Several peaks are observed, indicating regions with high flexibility. Notable peaks are found around residue positions 100, 200, 450, and 500. These regions exhibit RMSF values above 1.5 Å, suggesting significant movement during the simulation. The regions between the peaks. particularly around residue positions 0–100, 100–200, and 200–400, show lower RMSF values, often below 1.0 Å. These areas are relatively stable and do not undergo significant conformational changes. Both the N-terminal (residues 0–100) and C-terminal (residues 400–500) ends exhibit higher RMSF values (Figure 7) which is common as terminal regions are often more flexible compared to the core of the protein.

**FIGURE 8:**
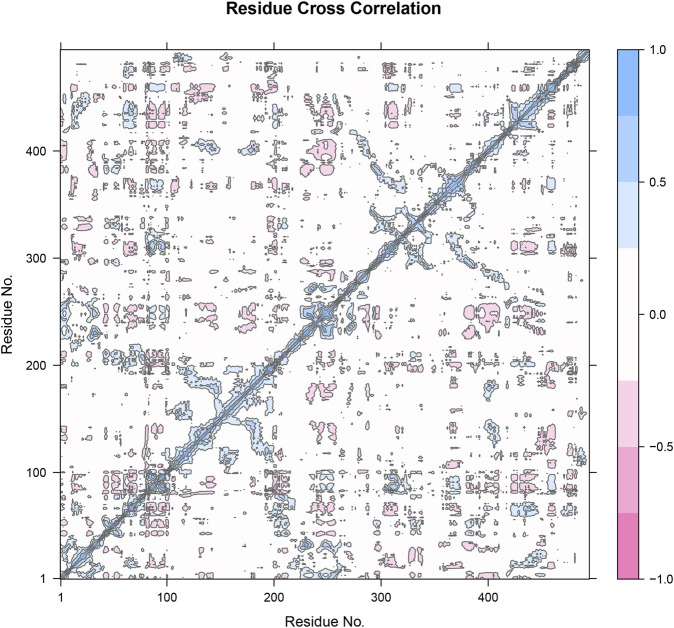
Residue cross-correlation matrix of compound 31.j3 in complex with the target protein (PDB ID: 3PO7) derived from molecular dynamics simulations.

The residue cross-correlation matrix obtained from the molecular dynamics simulation of Compound 31.j3 in complex with the target protein (PDB ID: 3PO7). The matrix displays the correlated motions between pairs of residues, with the xaxis and *y*-axis representing the residue numbers. The color scale on the right indicates the correlation coefficients, ranging from −1.0 to 1.0. Blue (1.0) indicates highly correlated motions, meaning the residues move in the same direction. Pink (−1.0) indicates highly anti-correlated motions, meaning the residues move in opposite directions. White (0) represents uncorrelated or independent motions The strong blue diagonal line (correlation coefficient of 1.0) indicates the self-correlation of residues, which is expected as a residue is always perfectly correlated with itself. Patches of blue and pink off-diagonal elements indicate regions of correlated and anti-correlated motions, respectively. Clusters of blue patches around residue numbers 100, 200, 300, and 400 suggest regions where groups of residues move in a concerted manner. These regions might correspond to secondary structural elements like alpha-helices or beta-sheets that undergo collective motions. Pink patches indicate residues that exhibit opposite directional movements (Figure 8). These anti-correlated motions could be indicative of hinge points or flexible regions that allow for conformational changes in the protein.

**FIGURE 9:**
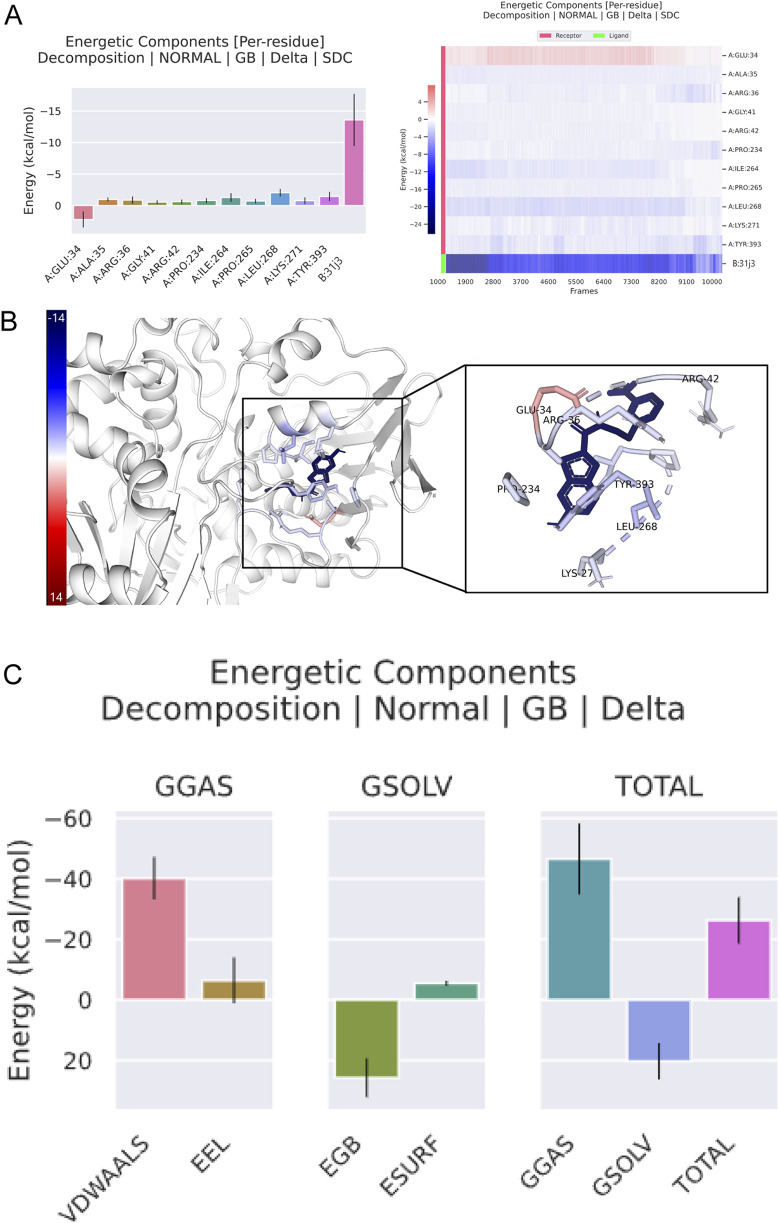
Energetic components analysis of compound 31.j3 in complex with target protein (PDB ID: 3PO7) from molecular dynamics simulation. **(A, B)** Compound 31.j3 Binding Pose with MAO-B Target; **(C)** Key Binding Interactions of Compounds 31 and 31.j3 with MAO-B.

Figure 9A shows the energetic contributions of individual residues to the total binding energy. Each bar represents a different residue, with energy contributions in kcal/mol. Certain residues, such as A-GLU-34, A-ALA-35, A-ARG-36,A-GLY-41, A-ARG-42, A-PRO-234, A-ILE-264, A-PRO-265, A-LEU-268, A-LYS-271, and A-TYR-393, exhibit significant contributions to the binding energy. Residues A-GLU-34 and A-ARG-36 show the highest contributions, indicating they play crucial roles in stabilizing the binding of compound 31.j3.

Figure 9B provides a visual representation of the target protein, highlighting the key residues that contribute significantly to the binding energy. The residues identified in Figure 8B are shown in the protein structure, illustrating their spatial arrangement and interaction with compound 31.j3. The highlighted residues ate. lecated.in close proximity to the binding site of compound 31.j3, reinforcing their importance in stabilizing the complex.

Figure 9C breaks down the total binding energy into various components: Van der Waals VDWAALS), electrostatic energy, polar solvation energy, and non-polar solvation energy. Each component’s contribution to the overall binding energy is represented in separate bar graphs. Van der Waals interactions contribute significantly to the binding energy, indicating strong hydrophobic interactions between compound 31.j3 and the target protein. Electrostatic interactions also play a crucial role, contributing positively to the binding stability. Solvation energies (both polar and nonpolar) are important for overall binding, with polar solvation energy showing a substantial positive contribution. The total binding energy is a combined result of these interactions, emphasizing the importance of a balanced contribution from different energetic components for stable binding. The energy breakdown of amino acid residues is shown in Table 7, with a total energy of −26.25 kcal/mol.

**TABLE 7:** Energy breakdown values of amino acid residues.

Frames	VDWAALS	EEL	EGB	ESURF	GGAS	GSOLV	Total
Average	−40.15	−6.38	25.73	−5.46	−46.52	20.27	−26.25
SD	6.91	7.5	6.37	0.75	11.72	6.0	7.61
SEM	0.07	0.08	0.07	0.01	0.12	0.06	0.08

The energetic components analysis of compound 31.j3 in complex with the target protein (PDB ID: 3PO7) reveals critical insights into the binding interaction. Key residues contributing significantly to the binding energy have been identified, with visual representation highlighting their importance in stabilizing the complex. Decomposition of the binding energy into its components shows that Van der Waals interactions, electrostatic interactions, and solvation energies all play vital roles in maintaining the stability and efficacy of compound 31.j3 simulations, and energetic analyses offers a robust platform for the rational design and optimization of new therapeutic agents targeting CRC. Future research will focus on further optimizing these inhibitors and experimentally validating their efficacy in biological systems, ultimately contributing to the development of more effective treatments for neurodegenerative diseases.

### 3.8 Mechanistic analysis of binding capabilities and stability

The RMSD analysis revealed that compound 31.j3 exhibits stable binding to the MAO-B receptor, with RMSD values fluctuating between 1.0 and 2.0 Å throughout the simulation (Figure 6). This stability can be attributed to several factors. First, the compound forms hydrogen bonds with key amino acid residues such as VAL-235, ALA-35, and GLU-34 (Table 6), which anchor the compound within the binding pocket and reduce its mobility. Second, the hydrophobic interactions with residues like GLY-16, GLY-13, and SER-15 (Table 6) further stabilize the compound by providing favorable van der Waals interactions (Figure 9C).

The energy decomposition analysis further supports these observations. The significant contributions of van der Waals interactions and electrostatic interactions to the total binding energy (Table 7) indicate that these non-covalent forces play crucial roles in stabilizing the complex. Specifically, residues like A-GLU-34 and A-ARG-36 exhibit the highest energy contributions, highlighting their importance in the binding process (Figures 9A, B).

Together, these mechanistic insights provide a comprehensive understanding of why compound 31.j3 exhibits superior binding capabilities and stability. The combination of hydrogen bonding, hydrophobic interactions, and favorable energetic contributions from key amino acid residues results in a stable and potent MAO-B inhibitor.

## 4 Discussion

### 4.1 Discussion of experimental results and future research directions

For Table 1: “The predicted IC50 values listed in the table were calculated by a 3D-QSAR model we constructed to rapidly assess the potential of the newly discovered 6-hydroxybenzothiazole-2-carboxamide derivatives as MAO-B inhibitors. The accuracy and reliability of the QSAR model can be verified by comparing it with experimentally measured IC50 values.

In addition to predicting the pIC50 values of the novel derivatives using the QSAR model, we further evaluated their performance through molecular docking and molecular dynamics simulations. Compound 31.j3 not only exhibited the highest predicted IC50 value but also scored the highest in molecular docking, with a total score of 8.1522 (Table 4). Molecular dynamics simulations revealed that compound 31.j3 maintained stable binding to the MAO-B receptor, with RMSD values fluctuating between 1.0 and 2.0 Å (Figure 6). Moreover, energy decomposition analysis showed that key amino acid residues, such as VAL-235, ALA-35, and GLU-34, contributed significantly to the binding energy of compound 31.j3 through hydrogen bonding and hydrophobic interactions (Table 6; Figure 9).

In this paper, we have selected 36 novel 6-hydroxybenzothiazole-2-carboxamide derivatives for our study, a choice based on several considerations.

These compounds are structurally diverse, especially in the amide substituent portion where multiple modifications have been made to introduce different side chains. This structural diversity provides us with a rich dataset that helps to explore the relationship between structure and activity. At the same time, these compounds exhibit a high degree of specificity in the inhibition of MAO-B, which is crucial for the development of targeted therapeutic agents against neurodegenerative diseases.

Prior to the selection of these compounds, we performed extensive pre-experimental screening, including preliminary enzyme activity tests and structure-activity relationship analyses. The results of these experiments indicated that these compounds showed good potential in inhibiting MAO-B. In particular, compound 31, as a reference molecule, showed the highest pharmacological activity in the experiments. Therefore, we used it as a basis to derive more compounds by structural modification in order to further explore the space for activity optimization.

We made theoretical predictions of the activities of these compounds using quantitative structure-effect relationship (QSAR) models. By COMSIA method, we constructed 3D-QSAR and successfully predicted the IC50 values of these compounds. To verify the accuracy of the theoretical predictions, we compared the predicted results with the actual experimental data and found good agreement between them. This further confirmed the structural rationality and predictability of our selected compound set in terms of activity.

The superior performance of compound 31.j3, as evidenced by its highest predicted IC50 value, molecular docking score, and stable binding in molecular dynamics simulations, comprehensively demonstrates its advantages over existing MAO-B inhibitors. The strong hydrogen bonding and hydrophobic interactions with key amino acid residues further support the robustness of its binding to the MAO-B receptor. These findings not only validate the efficacy of our QSAR model but also underscore the potential of compound 31.j3 as a promising candidate for the development of novel therapeutic drugs for neurodegenerative diseases.

These novel 6-hydroxybenzothiazole-2-carboxamide derivatives were selected for study not only because of their potential pharmacological activities, but also because of their structural novelty. This helps us to develop novel drugs with our own intellectual property rights. In addition, in view of the important role of MAO-B inhibitors in the treatment of neurodegenerative diseases, these compounds are expected to be important candidates for future drug discovery and have a broad application prospect.

### 4.2 Discussion of mechanistic insights

The mechanistic analysis presented here underscores the importance of considering multiple molecular interactions when designing potent and stable MAO-B inhibitors. The stable binding of compound 31.j3, as evidenced by the RMSD analysis, is the result of a well-orchestrated interplay between hydrogen bonding, hydrophobic interactions, and favorable energetic contributions.

In particular, the formation of hydrogen bonds with key amino acid residues within the MAO-B binding pocket appears to be a critical factor in stabilizing the compound. These hydrogen bonds not only anchor the compound but also facilitate the formation of favorable van der Waals interactions with nearby hydrophobic residues. The significant contributions of van der Waals and electrostatic interactions to the total binding energy further emphasize the importance of these non-covalent forces in stabilizing the complex.

By gaining a deeper understanding of the underlying mechanisms that govern the binding capabilities and stability of our compounds, we can more effectively guide the rational design of novel therapeutic agents targeting MAO-B. These insights provide valuable guidance for future drug discovery efforts aimed at developing potent and stable MAO-B inhibitors for the treatment of neurodegenerative diseases.

## 5 Conclusion

Through a broader comparison encompassing IC50 values, molecular docking scores, molecular dynamics simulations, binding free energies, and key amino acid residue contributions, we have demonstrated that the newly designed compound 31.j3 exhibits superior performance as a potential MAO-B inhibitor. Its efficient inhibitory activity, stable binding to the MAO-B receptor, and favorable interactions with key amino acid residues collectively support its development as a promising therapeutic agent for neurodegenerative diseases.

Based on the current results, we will further optimize the structure of compound 31.j3 to improve its bioavailability and selectivity, and validate its actual efficacy through synthesis and bioactivity tests. We will utilize more advanced biochemical and cell biological methods, such as proteomics, metabolomics and gene editing technologies, to fully reveal the mechanism of the protective effects of compound 31.j3 and its derivatives on neuronal cells. After confirming the safety and efficacy of the compounds, animal experiments and preclinical studies are advanced to assess their efficacy, pharmacokinetic properties and toxicity in the *in vivo* environment, providing an important basis for potential clinical applications. Given the complex and multifactorial nature of neurodegenerative diseases, explore the development of drugs that simultaneously target multiple targets, such as combining MAO-B and other enzymes or receptors associated with neurodegenerative diseases, to provide a more comprehensive neuroprotective effect.

## Data Availability

The original contributions presented in the study are included in the article/supplementary material, further inquiries can be directed to the corresponding authors.
